# Pilonidal Sinus: Open Surgery or PEPSiT? Our Preliminary Experience in Adolescents

**DOI:** 10.3390/children13030433

**Published:** 2026-03-22

**Authors:** Fabiola Cassaro, Salvatore Arena, Santi D’Antoni, Pietro Impellizzeri, Carmelo Romeo

**Affiliations:** 1 Unit of Pediatric Surgery, Department of Human Pathology of Adult and Childhood “Gaetano Barresi”, University of Messina, 98121 Messina, Italy; fabiola.cassaro@studenti.unime.it (F.C.); santi.dantoni@studenti.unime.it (S.D.); impellizzerip@unime.it (P.I.); romeoc@unime.it (C.R.); 2 Department of Biomedical and Dental Sciences and Morpho-Functional Imaging, University of Messina, 98124 Messina, Italy

**Keywords:** pilonidal sinus, endoscopic pilonidal sinus treatment, PEPSiT, minimally invasive surgery, fistuloscope

## Abstract

**Highlights:**

**What are the main findings?**

- Pediatric Endoscopic Pilonidal Sinus Treatment (PEPSiT) was associated with significantly shorter hospital stay, faster wound healing, and lower overall postoperative complication rates compared with traditional open excision in children and adolescents with pilonidal sinus disease.
- Recurrence rates were numerically lower after PEPSiT (9.5%) than after open surgery (25%), with comparable operative time between the two techniques.

**What are the implications of the main findings?**

- PEPSiT represents a safe and feasible minimally invasive alternative to open surgery in the pediatric population, aligning with the need for faster recovery and reduced wound-related morbidity in adolescents.
- Prospective, controlled, multicenter pediatric studies are needed to confirm long-term outcomes and to establish standardized treatment algorithms for pilonidal sinus disease in children

**Abstract:**

Background: Pilonidal sinus disease (PSD) is a chronic inflammatory condition commonly affecting the sacrococcygeal region, particularly in adolescents and young adults. Traditional open surgical approaches are associated with prolonged recovery, high complication rates, and recurrence. The advent of endoscopic techniques, such as Pediatric Endoscopic Pilonidal Sinus Treatment (PEPSiT), offers a promising alternative, reducing discomfort and potentially improving outcomes. The aims of the study were to compare the effectiveness and safety of PEPSiT versus traditional open excision in the treatment of PSD in the pediatric population. Methods: A retrospective, non-randomized study was conducted on patients aged 8–18 years who underwent surgery for PSD between 2019 and 2023 at our institution. Patients were divided into two groups: those undergoing traditional open excision (Group A) and those who received PEPSiT (Group B). Data were extracted from electronic medical records, including patient demographics, operative time, length of hospital stay, and post-operative complications such as recurrence and wound dehiscence. A minimum follow-up of 12 months was required. Statistical analysis was performed using the Chi-square test for categorical variables and Mann–Whitney U test for quantitative analysis. Results: A total of 61 patients were included in the study, with 40 undergoing Open surgery and 21 treated with PEPSiT. Mean operative time was shorter in the PEPSiT group (37.95 ± 10.86 min) compared with the Open group (47.85 ± 20.03 min), although this difference did not reach statistical significance (*p* = 0.052). Length of hospital stay was significantly reduced in the PEPSiT group (5.9 ± 8.73 h) compared with the Open group (15.40 ± 12.54 h) (*p* < 0.001). Post-operative complications were significantly less frequent following PEPSiT, with no cases of wound dehiscence observed compared with 27.5% in the Open group (*p* = 0.008). Recurrence rates were lower in the PEPSiT group (9.5%) than in the Open group (25%); however, this difference was not statistically significant (*p* = 0.149). Conclusions: PEPSiT is a feasible minimally invasive option for pediatric pilonidal sinus disease, associated with shorter hospital stay, faster wound healing, and fewer postoperative complications compared with open surgery, with comparable operative time. These results should nevertheless be interpreted with caution and warrant confirmation in prospective controlled studies.

## 1. Introduction

Pilonidal sinus disease (PSD) is a chronic and inflammatory condition that typically occurs in the sacrococcygeal region, and its development is strongly associated with obesity, deep gluteal cleft, hirsutism, local trauma, family history, sedentariness, and the presence of hair in the intergluteal cleft [1,2]. PSD is a frequent condition in adolescents and young adults, with a peak incidence during puberty and late adolescence. In pediatric populations, PSD represents a distinct clinical entity compared with adult disease, owing to differences in anatomical development, hormonal milieu, hair characteristics, lifestyle factors, and psychosocial impact [3]. These age-related features influence both disease presentation and treatment expectations and must be considered when evaluating surgical strategies. There is no single treatment pathway that has been proven to consistently provide definitive cure; therefore, clinicians must recognize the challenges associated with this condition and work collaboratively with the patient to develop an individualized treatment strategy [4]. The treatment for pilonidal sinus remains surgical, and various techniques have been described [5]. The goals of the ideal surgical technique should ensure the eradication of the cyst and thorough removal and cleaning of primary and secondary sinus tracts.

Traditional open surgical techniques, including wide excision with secondary healing or flap-based procedures, have long been considered the standard approach for PSD. In children and adolescents, these approaches are associated with prolonged healing, significant disruption of daily activities, and a non-negligible psychological burden [6,7,8].

Over the last decade, minimally invasive techniques, particularly endoscopic approaches [9] such as pediatric endoscopic pilonidal sinus treatment (PEPSiT), adapted for pediatric patients by Esposito in 2018, have gained increasing attention in pediatric surgery [10].

The aim of the study is to report our single-center experience, comparing the feasibility and safety of traditional open surgery versus PEPSiT for the treatment of PSD in the pediatric population, analyzing the incidence of complications, operative time, and length of hospital stay.

## 2. Materials and Methods

### 2.1. Study Population

Following approval by the Ethics Committee of the AOU “G. Martino” di Messina (Italy) (Prot. N. 11-25 AOU G. Martino, 11 March 2025), an observational retrospective comparative cohort study was conducted, including all patients who underwent surgery for PSD at our institution between 2019 and 2023. The study population included patients aged 8–18 years who were treated in the Pediatric Surgery Unit. Patients were classified into two groups: those who underwent traditional open surgery and those who received the PEPSiT procedure. Treatment allocation was not randomized but primarily time-based. Specifically, open surgery was routinely performed until 2021, whereas PEPSiT became the standard surgical approach in our unit starting from 2022. Therefore, the comparison reflects a before-and-after design related to the institutional adoption of the endoscopic technique rather than patient-specific selection criteria. All surgical procedures were performed within a tertiary-level Pediatric Surgery Unit. Both traditional open surgery and PEPSiT were carried out by experienced pediatric surgeons or by trainees under the direct supervision of a senior consultant, in accordance with routine clinical practice at our institution. Follow-up data were collected retrospectively from electronic medical records and follow-up visits to ensure consistent post-operative monitoring, with a minimum follow-up period of 12 months. Patients who were managed non-operatively or had incomplete clinical records were excluded from the study.

Collected data included patient demographics, operative time, incidence of recurrence and wound dehiscence, hospital readmission rates and length of hospital stay.

Complete healing was defined as full epithelialization of the surgical area, with absence of pain, discharge, signs of inflammation, or infection, as confirmed during scheduled outpatient follow-up visits. Recurrence was defined as the reappearance of chronic discharge, a sinus opening, abscess formation, or clinically evident pilonidal disease occurring after documented complete healing, and requiring further medical or surgical treatment or being confirmed on clinical examination during follow-up. Wound dehiscence has been considered as a partial or complete separation of sutured wound edges, applicable only to the open surgery group. Length of hospital stay was recorded in hours to allow a more precise quantification of postoperative hospitalization.

Recurrence timing was determined on the basis of clinical follow-up information.

Time to recurrence was calculated as the interval between the index procedure and the first clinical visit at which recurrence was identified.

When the exact date of recurrence could not be reliably established from the clinical records, the timing of recurrence was defined as the date of required surgical reintervention.

Patients who did not develop recurrence during the observation period were considered censored at the time of their last documented clinical follow-up.

Due to the retrospective nature of the medical record review, other postoperative complications could not be identified and were not considered in the analysis due to missing data and inconsistencies in follow-up. Patients were routinely evaluated 3 days after surgery and subsequently on a weekly basis until complete healing was achieved. After documented complete healing, patients entered periodic outpatient follow-up to monitor for recurrence. Follow-up was continued until complete healing and/or occurrence of recurrence, with a minimum follow-up duration of 12 months.

### 2.2. Statistical Analysis

Statistical analysis was conducted through frequency calculations and a descriptive analysis of the variables. Quantitative variables were expressed as mean ± standard deviation (SD), while non-quantitative variables were reported as absolute frequencies and percentages. Statistical analysis was performed using the Chi-square test for categorical variables and the Mann–Whitney U test for continuous variables. Time to first recurrence was analyzed using the Kaplan–Meier method. In order to account for potential differences in follow-up duration between the two groups, an additional Kaplan–Meier analysis restricted to the first 12 months of follow-up was performed. All analyses were carried out using IBM SPSS Statistics for Windows, Version 25.0 (Armonk, NY, USA: IBM Corp.). The threshold for statistical significance was set at *p* < 0.05.

### 2.3. Surgical Procedure

**Figure 1:**
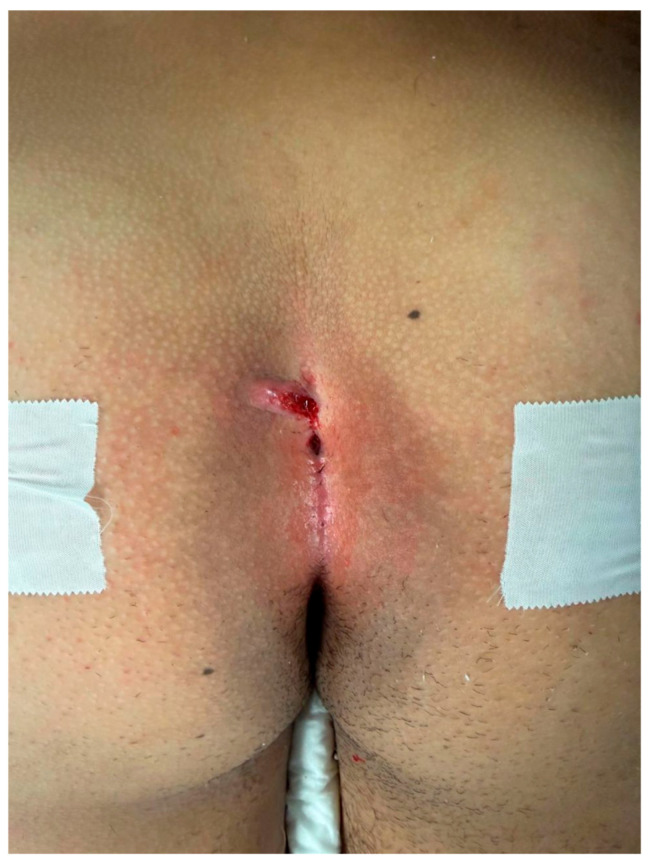
Gluteal cleft with multiple sinus tracts.

The patient position was the same for both surgical techniques: the prone position with external traction of both glutei to optimize surgical field exposure (Figure 1).

#### 2.3.1. Open Excision Surgery

An en bloc excision was performed by making an elliptical incision along the intergluteal midline, which included the cyst and all its fistulous tracts. The entire tissue involved was resected until the sacrococcygeal fascia was reached, and careful hemostasis was carried out. A drainage is placed in the wound cavity. The skin and subcutaneous tissue were then sutured with interrupted stitches.

#### 2.3.2. Endoscopic Surgery (PEPSiT)

**Figure 2:**
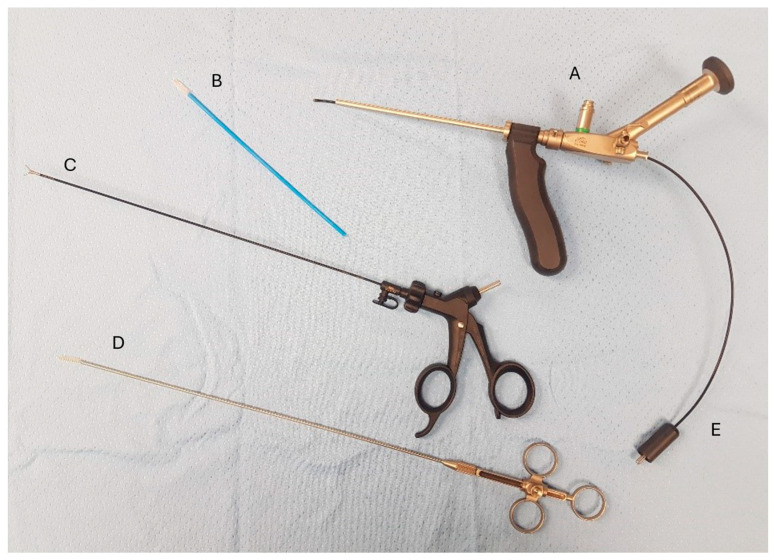
Instruments used for PEPSiT (A) Fistuloscope. (B) Fistula brush. (C) Forceps. (D) Fistula endobrush. (E) Monopolar electrode.

**Figure 3:**
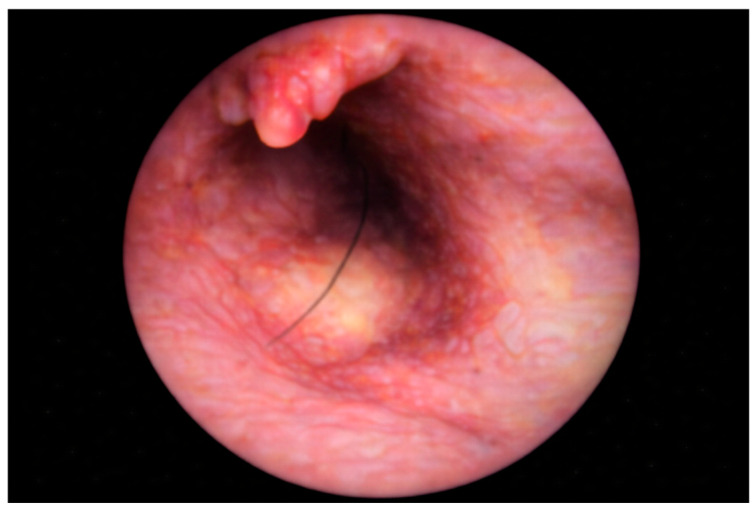
Endoscopic view at the sinus exploration.

**Figure 4:**
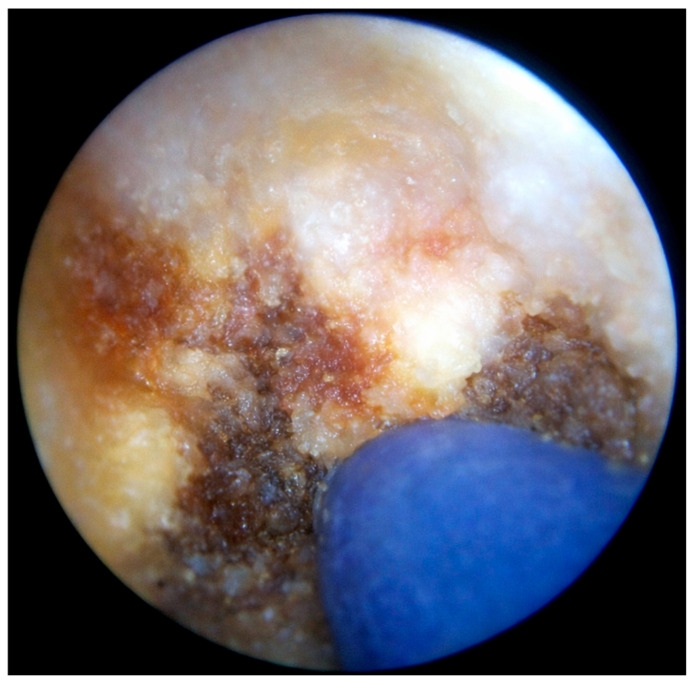
Thermocoagulation of the cavity’s wall using the endoscopic monopolar electrode.

**Figure 5:**
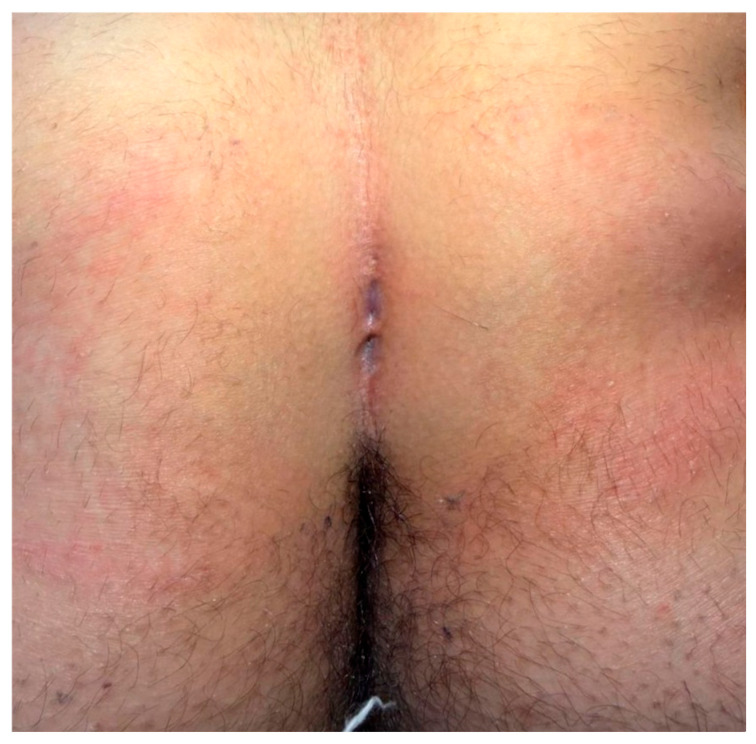
External appearance of the sinus at the end of PEPSiT procedure.

PEPSiT was performed either under regional anesthesia. The instruments used for PEPSiT include the fistuloscope (Karl Storz ^®^ GmbH—Tuttlingen, Germany), which is 18 cm long with a diameter of 3.3 × 4.7 mm. It features an 8° scope and an irrigation/working canal that allows instruments up to 2.5 mm in diameter to be introduced (Figure 2). After ensuring adequate asepsis and proper positioning of the operating field, the procedure starts by dilating one of the fistulous tracts using a curved mosquito forceps or a 0.5 cm circular incision made with a biopsy punch. The fistuloscope is then advanced through the dilated fistula (Figure 3). A continuous jet of mannitol solution is used to maintain a clear visual field during the procedure. The instruments allow the identification of the main sinus and possible secondary tracts. Then, the hairs have been removed with an endoscopic grasper introduced through the fistuloscope working channel. Electrofulguration of the pilonidal sinus and its fistulous tracts is carried out using the endoscopic monopolar electrode (Figure 4). The brush is employed for mechanical abrasion of the fistulas and cyst walls to remove necrotic material. Any remaining hair is extracted using the fistuloscope’s forceps under endoscopic vision. Wound care involves applying a stream of iodinated povidone to the surgical wound, allowing it to penetrate the tissue (Figure 5). In the postoperative period, wound care protocols differed according to the surgical technique and were standardized within each group. After open excision surgery, patients or caregivers were instructed to keep the wound dry and to perform daily local wound care using antiseptic solution (iodinated povidone), according to institutional practice, until complete healing. After PEPSiT, patients were managed at home with twice-daily irrigation of the small external opening using 5 mL of sterile saline solution until wound closure. No additional antiseptic agents were routinely used in this group.

### 2.4. Post-Operative Management

Post-operative pain management was obtained with oral paracetamol on demand. Post-operative antibiotics are not indicated [5,11]. Patients were routinely scheduled for follow-up visits after surgery to monitor wound healing and detect any early complications. Laser hair removal post-surgery, once full healing has been achieved, is recommended for all patients. After complete wound healing, patients were not routinely scheduled for long-term outpatient visits but were advised to return in case of symptoms suggestive of recurrence or complications. Therefore, follow-up after healing was based on a return-on-recurrence model rather than on structured, time-based assessments.

## 3. Results

A total of 61 patients underwent pilonidal sinus surgery at our Institution from January 2019 to December 2023. Of these, 40 patients underwent open surgery (Group A) between 2019 and 2021, while 21 patients underwent the PEPSiT procedure (Group B) between 2022 and 2023. The mean age at the time of surgery was 14.72 years ± 2.13 (10–18 years) with 55% of the cases being male (n = 33). The PEPSiT group had a mean age of 13.90 ± 2.26 years, while the Open group was slightly older (mean age = 15.59 ± 1.96 years) (Table 1).

Age differed significantly between the two groups, with patients in the PEPSiT group being younger than those in the open surgery group (Mann–Whitney U test, *p* = 0.037). Sex distribution did not differ significantly between groups (Fisher’s exact test, *p* = 0.529).

Surgeon experience was categorized as consultant surgeons with more than 5 years of experience or senior trainees under direct supervision. Among patients undergoing PEPSiT procedures were performed by consultants in 12 cases (57.1%) and by trainees in 9 cases (42.9%). In the open group, consultants performed 18 procedures (45.0%), while trainees performed 22 procedures (55.0%). No statistically significant difference in surgeon experience distribution was observed between open surgery and PEPSiT (χ^2^ = 0.81, *p* = 0.37; Fisher’s exact test *p* = 0.43).

The PEPSiT group demonstrated a mean operative time of 37.95 ± 10.86 min, with procedures completed in a range of 25 to 58 min. In contrast, the open group required longer surgical times, averaging 47.85 ± 20.03 min with a broader range of 28 to 85 min (*p* = 0.052).

The PEPSiT group demonstrated superior outcomes with no cases of abscess (0%) or dehiscence (0%), while the open group had significantly higher rates: 12 cases of abscess (12/40, 30%; *p* = 0.005) and 11 cases of dehiscence (11/40, 27.5%; *p* = 0.008). For recurrence, the PEPSIT group had 2 cases (2/21, 10%) compared to 10 cases in the open group (10/40, 25%), though this difference was not statistically significant (*p* = 0.149). A total of 61 patients were included in the analysis.

During the follow-up period, 12 patients (19.7%) experienced a first recurrence, while 49 patients (80.3%) remained recurrence-free and were censored at the time of last follow-up.

Kaplan–Meier analysis of time to first recurrence demonstrated a higher incidence and earlier occurrence of recurrence in the Open group compared with the PEPSiT group.

The median recurrence-free survival was not reached during the observation period, as fewer than 50% of patients experienced recurrence. Consequently, the estimated median survival corresponded to the maximum follow-up time of 36 months in both groups.

**Figure 6:**
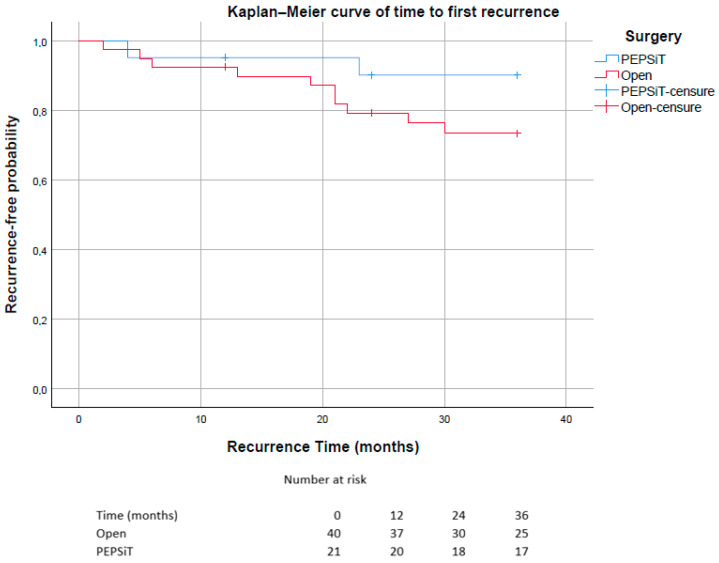
Kaplan–Meier curves showing time to first recurrence stratified by surgical technique (PEPSiT vs. Open surgery). Patients without recurrence were censored at the time of last follow-up. The number of patients at risk at selected time points (0, 12, 24 and 36 months) is reported below the graph.

In the PEPSiT group, recurrence events were infrequent, and the probability of remaining recurrence-free exceeded 90% throughout most of the observation period. In contrast, the open surgery group showed a progressive decline in recurrence-free probability, reaching approximately 73% at 36 months (Figure 6). The estimated mean recurrence-free time was 33.8 months in the PEPSiT group and 31.0 months in the open surgery group.

In the overall population, the estimated mean recurrence-free time was 31.9 months.

**Figure 7:**
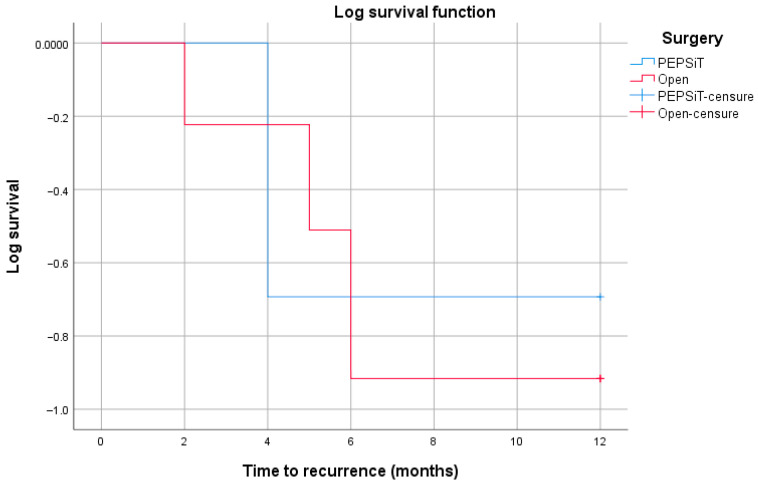
Kaplan–Meier curves of recurrence-free survival within the first 12 months of follow-up according to surgical technique (PEPSiT vs. open surgery). Censored observations are indicated by tick marks. No statistically significant difference between the groups was observed using the log-rank (Mantel–Cox) test (χ^2^ = 0.006, *p* = 0.937).

A log-rank (Mantel–Cox) test restricted to the first 12 months of follow-up did not show a statistically significant difference in recurrence-free survival between the PEPSiT and open surgery groups (χ^2^ = 0.006, *p* = 0.937) (Figure 7).

Post-operative complications overall were also assessed, combining abscess, dehiscence, and recurrence. By combining abscess, wound dehiscence, and recurrence, the overall complication rate was significantly higher in the open surgery group (35%, 14/40 patients) compared to the PEPSiT group (9.5%, 2/21 patients) (*p* < 0.001).

The PEPSiT group demonstrated a significantly shorter length of hospital stay (mean 5.90 ± 8.73 h) compared to the open group (mean 15.40 ± 12.54 h; *p* < 0.001). The healing process showed even more differences, with PEPSiT patients recovering in 28.67 ± 11.68 days versus 52.71 ± 18.28 days for open with a *p* < 0.001. All outcomes are shown in Table 2.

## 4. Discussion

This study presents a comparative analysis of open surgical procedures and PEPSiT in a pediatric and adolescent cohort treated at a single institution over a defined time period.

Pilonidal sinus disease is a common inflammatory condition affecting predominantly the pediatric and young adult population. Although its true incidence remains unclear, it has been estimated at approximately 2.63 per 1000 patients are affected [12] PSD predominantly affects males, with a male-to-female ratio of 2-3:1, and is frequently associated with a positive family history [1,12]. While the exact pathogenesis remains incompletely understood; however, factors such as genetic predisposition, mechanical irritation due to prolonged sitting, obesity, poor hygiene, excessive perspiration, and excessive hair have been suggested to contribute [1]. Initial treatment should focus on modifiable risk factors, including hair removal, local hygiene, and avoidance of prolonged sitting, which should be maintained throughout the course of the disease. Non-invasive options such as laser epilation and phenol application may be effective in selected cases, although recurrence remains possible [13,14].

Traditional surgical approaches typically involve excision of the sinus tract, followed by either primary closure or healing by secondary intention. While primary closure offers the advantage of avoiding wound packing and enabling faster healing, it is associated with a higher risk of complications, particularly wound dehiscence. Literature reports indicate that conventional excision techniques frequently result in high complication rates, including partial or total wound dehiscence [15]. Pediatric and adolescent patients with pilonidal sinus disease exhibit higher and earlier recurrence rates compared to adults, particularly in those nearing adulthood [4]. Unlike adult populations, pediatric patients place particular emphasis on rapid functional recovery and minimal disruption to daily life. In this context, the reduced invasiveness of PEPSiT represents a relevant advantage. The shorter healing time and avoidance of large open wounds are especially meaningful in adolescents, for whom prolonged wound care may negatively impact school attendance, sports participation, and psychosocial well-being [16]. These pediatric-specific priorities are often underrepresented in adult-driven literature and justify a dedicated pediatric evaluation of minimally invasive approaches, in this setting, PEPSiT has emerged as a safe and effective treatment option for PSD [17].

In 2018, Esposito et al. adapted the technique for pediatric patients, introducing Pediatric EPSiT (PEPSiT). This approach showed promising results, including shorter operative time, reduced need for postoperative analgesia, shorter hospital stay, time to resume full daily activities, and lower rates of complications such as bleeding and recurrence [11]. A technical modification involving the use of a glycine/mannitol irrigation solution instead of saline was also introduced to improve visualization and operative field clarity. Since then, PEPSiT has been increasingly adopted by pediatric surgical units as a preferred treatment option for pediatric PSD.

Our experience is in agreement with these findings regarding length of stay and wound healing time, while operative time did not differ significantly between the two approaches. In our series the operative time did not differ significantly between the two techniques: the mean difference was approximately 9 min (47.85 ± 20.03 min for Open vs. 37.95 ± 10.86 min for PEPSiT), without a clear advantage for either approach.

Our study supports PEPSiT as an effective surgical technique for pediatric and adolescent patients. In our study cohort, one-third of patients who underwent open surgery experienced wound dehiscence, whereas no cases of wound dehiscence were observed in the PEPSiT group, as the technique does not require a formal wound closure. Additionally, only one patient in the PEPSiT group experienced recurrence, whereas open surgery was associated with a 25% recurrence rate. Recurrence often needs reoperation, leading to patient dissatisfaction. Moreover, given the etiopathogenesis of PSD, the elimination of hair follicles via laser hair removal may play a crucial role in reducing recurrence rates [18]. Finally, PEPSiT offers significant cosmetic advantages, which are highly valued by young patients, further highlighting its potential as a minimally invasive and effective treatment for PSD [18]. However, the guidelines highlight the scarcity of systematic reviews and emphasize the urgent need for high-quality randomized controlled trials to better define the role of these techniques in PSD management [5,19].

Despite the valuable insights provided by our study, certain limitations should be acknowledged. The retrospective and non-randomized design of the study limits data completeness, and the unequal sample sizes between the open surgery and PEPSiT groups may introduce selection bias. As a result, potential benefits related to quality of life, cosmetic outcomes, or return to daily activities cannot be directly derived from the present data. Time-to-event data for recurrence were not consistently available, precluding reliable survival analysis.

Although the adoption of the surgical approach in this series mainly followed a time-related change in institutional practice, patients undergoing PEPSiT were, on average, younger than those treated with open surgery. This difference in age indicates that the two groups may not be fully comparable. In this context, age-dependent factors such as pubertal development and hair characteristics could have varied between cohorts and may have contributed to differences in disease presentation and outcomes, irrespective of the surgical technique employed.

The limited availability of pediatric-specific comparative data represents an ongoing challenge in the management of pilonidal sinus disease and underscores the need for dedicated pediatric studies rather than extrapolation from adult populations.

Taken together, these findings support the role of PEPSiT as a valuable minimally invasive option in pediatric PSD, offering advantages aligned with the priorities of adolescent patients. Nevertheless, our results should not be interpreted as definitive evidence of superiority over open surgery. Rather, they highlight the need for standardized pediatric-specific outcome measures, detailed reporting of disease severity and surgeon experience, and structured follow-up protocols.

Further prospective, multicenter studies with larger cohorts are warranted to confirm these findings and to establish standardized treatment algorithms for pediatric and adolescent PSD.

## 5. Conclusions

In this single-center retrospective cohort of pediatric patients with pilonidal sinus disease, PEPSiT was associated with significantly shorter hospital stay and faster wound healing compared with traditional open surgery. The endoscopic approach also showed a lower rate of postoperative complications, while operative time was comparable between the two techniques. Although recurrence rates were numerically lower in the PEPSiT group, this difference did not reach statistical significance. These findings suggest that PEPSiT represents a safe and effective minimally invasive option for the treatment of pilonidal sinus disease in children and adolescents. Nevertheless, larger prospective multicenter studies are required to confirm these results and to better define the role of PEPSiT in pediatric surgical practice.

## Figures and Tables

**Table 1:** Demographic characteristics. M: male, F: female.

	Open Surgery (Group A)	PEPSiT (Group B)	Total
Patients (n)	40 (65.6%)	21 (34.4%)	61
Age (years, mean)	15.59 ± 1.96	13.90 ± 2.26	14.72 ± 2.13
Sex (n)	M 22 (55%)	M 11 (52.4%)	M 33 (55%)
	F 18 (45%)	F 10 (47.6%)	F 28 (45%)

**Table 2:** Outcomes Measures (* *p*-value statistically significant).

	Open Surgery (Group A)	PEPSiT (Group B)	*p*-Value
Post-operative complications (n)	14 (35.0%)	2 (9.5%)	<0.001 *
Abscess	12 (30.0%)	0 (0.0%)	0.005 *
Wound dehiscence (n)	11 (27.5%)	0 (0%)	0.008 *
Recurrence (n)	10 (25.0%)	2 (9.5%)	0.149
Operative time (minutes)	47.85 ± 20.03	37.95 ± 10.86	0.052
Length of stay (hours)	15.40 ± 12.54	5.9 ± 8.73	<0.001 *
Wound healing (days)	52.71 ± 18.28	28.67 ± 11.68	<0.001 *

## Data Availability

The original data presented in the study are openly available in https://1drv.ms/f/c/6b5c03768417e272/IgAeie2s1sYaSYao-krHYmXYAW8fFWX_RnLuOLX5IhLcj50?e=ecvpr7 (accessed on 4 March 2026).

## Abbreviations

The following abbreviations are used in this manuscript:

PSD	Pilonidal Sinus Disease
SPSD	Sacrococcygeal Pilonidal Sinus Disease
PEPSiT	Pediatric Endoscopic Pilonidal Sinus Treatment
EPSiT	Endoscopic Pilonidal Sinus Treatment
MIS	Minimally Invasive Surgery
